# Zolpidem-related euphoria, addiction and detoxification: A case report and review of the literature

**DOI:** 10.1097/MD.0000000000040280

**Published:** 2024-11-01

**Authors:** Fangfei Xie, Bo Liu, Liqiu Yang, Junqiang Huang, Bin Li, Yuanyuan Li

**Affiliations:** a Mental Health Center and Psychiatric Laboratory, West China Hospital of Sichuan University, Chengdu, China; b Chengdushi Dekang Hospital, Chengdu, China; c Zigong Mental Health Center, Zigong, China.

**Keywords:** addiction, case report, detoxification, euphoria, sensation-seeking, Zolpidem

## Abstract

**Rationale:**

Zolpidem, a non-benzodiazepine sedative-hypnotic, is considered safer for the treatment of insomnia compared to benzodiazepines. However, in recent years, there have been growing reports of Zolpidem dependence, addiction, and withdrawal symptoms. We reported a case of Zolpidem addiction and successful detoxification, reviewed similar cases in the literature, and proposed a potential mechanism underlying Zolpidem addiction.

**Patient concerns:**

The patient was a 46-year-old Tibetan woman who had been using Zolpidem intermittently to treat insomnia for at least 8 years. She was overweight, with a BMI of 28.04 kg/m², and had hypertension, diabetes, a 20-year smoking history, and several years of alcohol abuse, often seeking instant gratification. She voluntarily increased both the dosage and frequency of Zolpidem, experiencing euphoria, anxiolysis, and increased appetite at higher doses, which led her to gradually escalate her dosage to 280 mg per day in divided doses. However, upon stopping Zolpidem, she experienced withdrawal symptoms, including insomnia, tension, and palpitations.

**Diagnoses:**

She was diagnosed with a combination of hypnotic use disorder, anxiety disorder, hypertension, and diabetes.

**Interventions:**

She underwent diazepam replacement therapy, along with antianxiety medications and mindfulness-based cognitive therapy, to address Zolpidem addiction.

**Outcomes:**

After 13 days of inpatient treatment, the patient successfully quit Zolpidem. During a 3-month follow-up, she returned to work and remained free from Zolpidem use.

**Lessons:**

We speculate that Zolpidem addiction is likely linked to the drug’s euphoric effects and certain patient characteristics, such as sensation-seeking behavior. A comprehensive approach, involving both pharmacological and psychological interventions, is essential for an effective detoxification strategy for Zolpidem addiction.

## 1. Introduction

Zolpidem, as a non-benzodiazepine sedative-hypnotic agent, was initially hailed as a safe alternative to benzodiazepines (BZDs) for short-term insomnia, as BZDs are known for their potential for dependency. However, there has been a growing number of reports indicating Zolpidem abuse, dependence, and withdrawal effects over time.^[1]^ Zolpidem dependence occurs when an individual long-term misuses the medication, resulting in drug tolerance and withdrawal symptoms, along with impaired function.^[2]^ Drug tolerance refers to the reduced effectiveness of a drug over time, necessitating higher doses to achieve the same desired effect, while withdrawal refers to the set of physiological and psychological symptoms that occur when a person abruptly stops or significantly reduces their use of a substance after prolonged or heavy use.^[3]^ Evidence from case reports indicates that individuals can have a wide range of tolerance for Zolpidem, with reported doses ranging from 10 to 6000 mg per day.^[4]^ It is reported that Zolpidem withdrawal symptoms mainly include seizures, tremors, delirium, sweating, palpitations, irritability, and other symptoms.^[4,5]^ Although Zolpidem prescription guidelines recommend use solely for severe insomnia and for short duration, employing the lowest effective dose possible,^[6]^ the misuse of Zolpidem outside of clinical supervision remains a prevalent issue, constituting a primary factor contributing to Zolpidem dependence and addiction.

Detoxification denotes the safe cessation of a substance of dependence, typically under medical supervision.^[7]^ Due to the absence of standardized clinical guidelines for Zolpidem detoxification, the reported approaches to addressing zolpidem addiction vary across different studies. The most commonly utilized method for Zolpidem detoxification involves substituting with long-acting BDZs, such as Diazepam^[8]^ or Clonazepam.^[4]^ In addition to pharmacological treatments, psychosocial therapies are essential for enhancing detoxification,^[7]^ as individuals with drug addiction often exhibit specific characteristics such as sensitivity, emotional instability, impulsiveness, and a tendency towards hedonism.

Here, we present an intriguing case of an 8-year zolpidem addiction with a 20-year tobacco history, successfully addressed through a detoxification regimen incorporating diazepam tapering, antianxiety medications, and mindfulness-based cognitive therapy (MBCT). In addition, we reviewed the available approaches for detoxification from Zolpidem and proposed a comprehensive assessment of the patient’s sensation-seeking traits and the risk of experiencing Zolpidem-related euphoria.

## 2. Case presentation

The patient was a 46-year-old Tibetan woman who had been experiencing intermittent insomnia for up to 20 years, particularly when feeling anxious or upset due to unpleasant life or work events. She struggled to fall asleep, woke up early, and experienced daytime depression and decreased work capacity. She typically used alcohol to help her sleep but did not seek medication. Eight years before her admission, she began taking a 10 mg dose of Zolpidem at bedtime to help with her worsening insomnia. Seeing improvement, she continued using the medication regularly for 2 years despite her sleep quality fluctuating with her unstable emotions. Later, she purposefully raised the dosage to 40 mg per night in pursuit of a pleasant state. She described feeling euphoria, having an increased appetite, and experiencing positive emotions after taking a high dose of Zolpidem.

**Figure 1.**
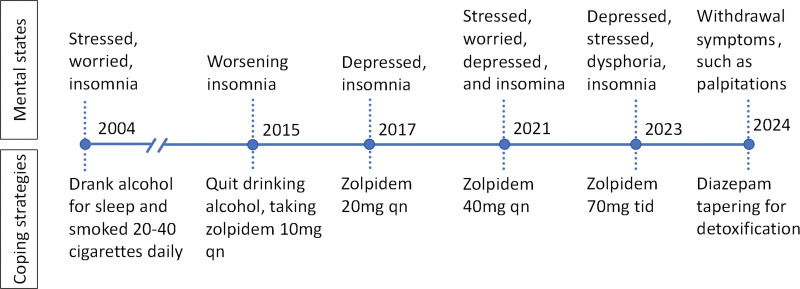
A brief timeline of the patient’s zolpidem addiction and detoxification. qn represents once per night, and tid represents their times per day.

On the contrary, her family members noted that she was prone to dysphoria and anger. Concerned about the adverse effects of Zolpidem, her family members advised her to reduce and stop taking the drug gradually. Due to poor sleep quality and a noticeably negative mood, she struggled to discontinue the medication and resorted to taking it secretly as she felt it was necessary. Two months before being admitted, she increased her daily dose of Zolpidem to 280 mg, divided into 3 doses. She mentioned feeling euphoric and pleasant when taking this medication but experienced tension, palpitations, and poor sleep when trying to discontinue it. During that period, the frequent use of high doses of Zolpidem significantly impaired her work performance, as she often felt drowsy and experienced decreased attention and memory. As a result, she was admitted to our hospital for Zolpidem detoxification, accompanied by her family members (see Fig. 1).

She was single and lived with her parents and brother, with no plans to marry. She exhibited a boyish demeanor, often seeking instant gratification. She had a 20-year history of smoking and several years of alcohol abuse. She reported smoking approximately 20 to 40 cigarettes per day but had given up alcohol many years ago. She worked as a civil servant in a town in Tibet and had good relationships with her colleagues and family. She tended to become anxious when confronted with daily tasks. Additionally, she had been diagnosed with both hypertension and diabetes a few years ago. Her blood pressure was controlled within the normal range, but her blood glucose levels fluctuated significantly, likely linked to her diet habits, lack of exercise, and anxiety.

On admission, the patient was 158 cm tall and weighed 70 kg, resulting in a 28.04 kg/m² BMI. Her vital signs were within the normal range. Physical examination revealed no apparent abnormalities. Mental examination showed significant symptoms of anxiety syndrome, such as dysphoria, upset, anticipatory anxiety and withdrawal syndrome, such as palpitations, tachycardia, tension, insomnia, and psychological craving for Zolpidem. Laboratory examinations revealed levels of random blood glucose at 13.12 mmol/L, urine glucose at 28 mmol/L, blood triglycerides at 1.92 mmol/L, and a white blood cell count of 13.79 × 10^9^/L (see Table 1).

**Table 1:** Laboratory examination results.

Test	Results
CBC	WBC: 13.79 × 10^9^/L↑, NEUT%: 81.7%↑
Blood biochemistry	GLU: 13.12 mmol/L↑, TG: 1.92 mmol/L↑, UA: 398 μmol/L↑
Blood gas analysis	PH: 7.309↓, LAC: 4.9 mmol/L↑
Blood eletrolytes	No abnormalities
Blood hormonal level	ACTH: 3.14 ng/L↓, T3: 0.75 nmol/L↓, FT3: 2.35 pmol/L↓
Urine analysis	GLU: 28 mmol/L↑, BACT: 6374/μL↑
Zolpidem-specific test	Not available in our hospital
ECG	Sinus bradycardia with arrhythmia
EEG	No abnormalities
Abdominal ultrasonography	Fatty liver
Chest CT	Scattered mild inflammation in both lungs
Brian MRI	No abnormalities

ACTH = corticotropin, BACT = bacteria, CBC = complete blood count, CT = computed tomography, ECG = electrocardiogram, EEG = electroencephalogram, FT3 = free triiodothyronine, GLU = glucose, LAC = lactic acid, MRI = magnetic resonance imaging, NEUT = neutrophile, T3 = triiodothyronine, TG = triglycerides, UA = uric acid, WBC = white blood cell.

**Figure 2.**
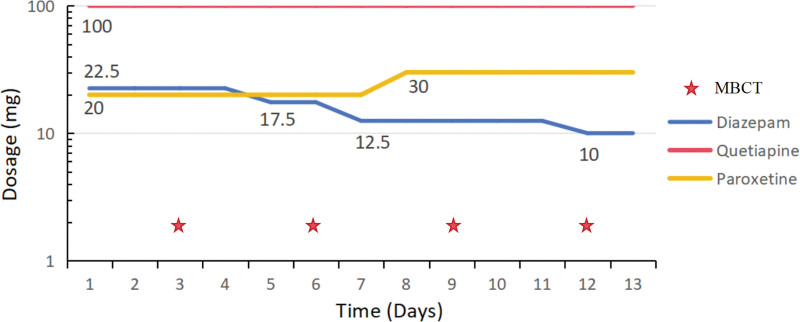
The detailed procedure of zolpidem detoxification of the patient. MBCT: mindfulness-based cognitive therapy, twice weekly. The diazepam was tapered from 22.5 mg to 10 mg per day, while paroxetine was titrated from 20 mg to 30 mg per day.

Additionally, the Hamilton Anxiety Scale score was 30 points. She was diagnosed with a combination of hypnotic use disorder, anxiety disorder, hypertension, and diabetes. She received a diazepam taper approach to address the Zolpidem withdrawal response and a titration approach with Quetiapine and Paroxetine for antianxiety treatment (see Fig. 2). She also takes Linagliptin-metformin 1 tablet twice a day, Dapagliflozin 10 mg daily to control blood glucose, and Valsartan-amlodipine 1 tablet once per day to control blood pressure. Furthermore, she also underwent MBCT twice a week, with a focus on addressing her addictive behavior and unstable emotions. After 13 days of hospitalization, her sleep had improved, and her anxiety symptoms had been alleviated without apparent adverse effects. Additionally, she reported that her blood glucose and blood pressure remained stable. Following discharge, we conducted regular monthly follow-ups and health education via phone. Her sleep and emotions remained stable, and she returned to work without using Zolpidem anymore.

## 3. Discussion

Zolpidem, Zaleplon, and Zopiclone, collectively referred to as Z-drugs, are a class of medications that exhibit agonistic effects on gamma-aminobutyric acid (GABA_A_) receptors. They are characterized by a shorter duration of action and half-life and fewer residual effects. In contrast to BZDs, they do not significantly modify the overall sleep architecture and are believed to be associated with a reduced risk of severe dependence, tolerance, and withdrawal symptoms.^[9]^ However, there is a growing body of reports documenting instances of Zolpidem abuse, dependence, and addiction.^[8]^ Almost all cases of Zolpidem addiction are attributed to off-label use by the patient. Interestingly, when Zolpidem dependence develops, long-acting BZDs can often be utilized as a substitution therapy.

Based on reported literature and clinical practice, there are several different detoxification approaches for managing Zolpidem dependence or addiction. For example: (1) The long half-life BZDs replacement approach, such as using diazepam or clonazepam, is commonly used to address the withdrawal symptoms of Zolpidem. This method includes 2 subtypes of substitute regimens: BZDs tapering^[4,8,10]^ or titrating^[11]^ while completely stopping Zolpidem. (2) The Zolpidem taper approach is suitable for mild withdrawal symptoms or special situations, such as during pregnancy.^[12]^ (3) A cross-titration regimen refers to gradually decreasing Zolpidem while simultaneously increasing BZDs.^[13]^ (4) Flumazenil, a GABA_A_ receptor antagonist, can reverse the effects of Zolpidem. A case series reported successful detoxification of Zolpidem using Flumazenil.^[14]^ Due to the significant adverse effects associated with Flumazenil, such as seizures, it is essential to carefully evaluate the benefit-to-risk ratio of administering Flumazenil in each patient experiencing an overdose.^[15]^ (5) Several clinical cases have utilized cholinesterase inhibitors, serving as nonspecific antagonists of the GABA_A_ receptor, in the detoxification process of Zolpidem.^[16]^ Furthermore, since the majority of patients exhibit mood disorders, other medications, including antianxiety, antidepressants, mood stabilizers, and antipsychotics, are used as supplementary treatments for the detoxification of Zolpidem. For example, other cases reported that patients with Zolpidem dependence were successfully detoxified using Gabapentin^[17]^ or Pregabalin.^[18]^ Here, we have listed a selection of representative case reports on the detoxification of Zolpidem from the last 20 years (see Table 2).

**Table 2:** Partial list of reports on zolpidem detoxication approaches from the last 20 years.

References	Year	Sex/age	Substances abuse history	Diagnosis	Maximum dose (mg/d)	Duration of misuse (month)	Sensation-seeking	Withdrawal symptoms	Detoxification agents and approaches
Moshfeghinia et al^[4]^	2023	F/39	Not given	Insomnia	6000	120	Euphoria	Seizure, body tremors, nystagmus, a nxiety, hot flashes, and sweaty palms	Clonazepam tapering Escitalopram Quetiapine Sodium-valproate
Awasthi et al^[5]^	2023	M/25	No	Stress-induced insomnia	70	10	Not given	Delirium, sweating, hallucinations, insomnia	Lorazepam tapering
Mao et al^[11]^	2022	M/25	Alcohol, cigarette, marijuana, nitrous oxide	Bipolar disorder	80	24	Relaxed	Anxiety, paresthesia, craving, influenza-like symptoms, seizures, and allucinations	Clonazepam titrating Olanzapine titrating Lithium titrating
Lyu et al^[10]^	2022	F/23	No	Zolpidem dependence, depression	1400	72	Euphoria	Tremor, sweating, palpitation, nausea, and dysphoria	Diazepam tapering Bupropion Mirtazapine
Bhatia et al^[12]^	2022	F/36	Not given	Affective disorder, pregnancy	300	144	Not given	Anxiety, restlessness, headache, and seizure	Zolpidem tapering
Bajaj et al^[19]^	2019	M/45	Alcohol	Zolpidem withdrawal, Zolpidem dependence	2400	60	Euphoria	Limb weakness, difficulty concentrating, dizziness, reduced sleep, and pronounced irritability	Clonazepam titrating Propranolol titrating
Sabe et al^[20]^	2019	F/47	No	Manic episode, Zolpidem dependence	160	36	Euphoria	Mania	Olanzapine Oxazepam
Chattopadhyay et al^[21]^	2016	M/33	No	Zolpidem dependence, Nicotine dependence	1700	60	Euphoria	Minor headaches and tremors	Not applicable
Lin et al^[16]^	2014	F/48	Not given	Major depression, Insomnia	400	36	Not given	Nausea, vomiting, and diarrhea	Galantamine Flurazepam Trazodone Quetiapine
Fernandes et al^[17]^	2013	F/72	Not given	Insomnia	300	8	Energetic	Irritable, decreased energy, tremor, craving	Gabapentin titrating Zolpidem tapering
Chen et al^[13]^	2012	F/53	No	Depression, Insomnia	160	48	Energetic	Palpitations, tremor, and anxiety	Zolpidem tapering Clonazepam titrating
Oulis et al^[18]^	2011	F/49	Not given	Dysthymic disorder	1500	60	Relaxed Energetic	Seizures	Pregabalin titrating Zolpidem tapering
Quaglio et al^[14]^	2005	M/38	Polydrug	Insomnia	900	24	Antianxiety	Trembling, sweating, nausea, irritability, anger, and agitation	Flumazenil titrating clobazam Sertraline
Quaglio et al^[14]^	2005	M/27	Cannabis, amphetamine, cocaine	Insomnia	1600	48	Antianxiety	Not given	Flumazenil titrating clobazam Sertraline
Rappa et al^[8]^	2004	M/46	Polysubstance	Zolpidem withdrawal, Zolpidem dependence	400	24	Antianxiety	Headache, tremors, shakiness, sweats, chills, and rebound insomnia	Diazepam tapering Atenolol Nefazodone

The experience of euphoria caused by Zolpidem is a significant factor leading to addiction to the medication. In our case, the patient reported feeling euphoric and excited after taking a high dose of Zolpidem. Similarly, X. Lyu et al reported that a 23-year-old female experienced mania-like symptoms, such as euphoria, increased activity, reckless behavior, constant talking and bragging, particularly when taking a high dose of Zolpidem on an empty stomach.^[10]^ Another case study^[21]^ documented a 33-year-old male who consumed 1700 mg of Zolpidem per day. He reported feelings of euphoria and increased talkativeness, which did not appear to impact his work or life. We reviewed cases of Zolpidem addiction from the past 20 years. We observed that at least 80% of reported cases described experiencing euphoria or a sensation of relaxation (see Table 2), similar to being intoxicated. Since alcohol primarily interacts with the GABA_A_ receptor in the brain,^[22]^ it is plausible that Zolpidem and alcohol share similar binding sites on this receptor, leading to comparable pharmacological effects.

The propensity for addiction often arises from behaviors aimed at pursuing euphoria or evading dysphoria. Euphoria, characterized by intense excitement and happiness, represents an amplification of pleasure and an experience of intense feelings of well-being.^[23]^ For instance, addiction to Opioids is closely linked to their potent analgesic properties and the elicitation of euphoric effects.^[24]^ Evidence from numerous case reports suggests that excessive use of Zolpidem is often motivated by a desire to seek euphoria,^[4]^ relaxation^[11]^ or antianxiety,^[14]^ in addition to its prescribed use for insomnia. This is evidenced by the fact that many patients take the medication during the daytime. In addition to patient psychological traits, such as sensation-seeking, the euphoric effects of Zolpidem may also contribute significantly to its misuse, dependency, and addiction.

Moreover, a recent systematic review indicates that Zolpidem has shown promise in treating various neurological disorders, particularly movement disorders and disorders of consciousness.^[25]^ These phenomena suggest that Zolpidem may have multiple pharmacological effects beyond its primary function of improving insomnia. The current understanding of Zolpidem pharmacodynamics indicates a selectivity for the α1 subtype of GABA_A_ receptors.^[26]^ However, at higher doses, Zolpidem may lose this selectivity and bind to other subtypes of GABA_A_ receptors.^[8]^ Additionally, the diverse distribution of GABA_A_ receptor subtypes, each consisting of distinct subunit compositions across the brain, may help explain the dose-dependent effects of Zolpidem.^[27]^

Despite long-term exposure to high doses of Zolpidem, with the highest dose up to 6000 mg per day,^[4]^ liver and renal function abnormalities have been rarely reported,^[4,19,28]^ which was also supported by our case. In contrast, the effects of Zolpidem on the brain are observable, including seizure, delirium, reckless behaviors, irritability, and cognitive impairments.^[29]^ These data suggest that Zolpidem may exert selective effects on the central nervous system compared to peripheral organs, likely due to differences in GABA_A_ receptor systematic distribution. Like its unexpected effects, it is essential to recognize that the adverse effects of Zolpidem on the central nervous system may extend beyond our current understanding.^[30]^ However, a large amount of evidence comes from clinical observation, and there are few randomized controlled trials to assess the emerging neurological effects and safety of Zolpidem.

Additionally, another significant factor contributing to Zolpidem addiction is the psychological susceptibility of the patient. Among the reported cases of Zolpidem addiction, a majority of the patients are sensation-seekers who seek instant gratification and exhibit a strong avoidance of discomfort, characteristics frequently linked to the development of substance addiction.^[31]^ In our case, the patient also exhibits instant gratification and leads an unhealthy lifestyle characterized by several detrimental habits, including a history of alcohol abuse, a 20-year smoking habit, a lack of exercise, and an unhealthy diet. These factors have contributed to her hypertension, diabetes, overweight condition, and unstable emotional state, as well as substance addiction. A history of substance abuse and psychosis are often considered important risk factors for developing Zolpidem addiction.^[32]^

To some extent, sensation-seeking behaviors can be attributed to a lack of wisdom or cognitive deficits. Education and cognitive behavioral therapy have demonstrated effectiveness in reducing long-term BZDs and Z-drug use.^[33]^ MBCT has been demonstrated to be effective in treating affect regulation issues that are specific to co-occurring addictive and mood disorders.^[34]^ In our case, the patient demonstrated the efficacy of MBCT in reducing cravings for Zolpidem.

Despite clinicians being aware of the potential for Zolpidem dependency and prescribing it strictly by instructions, the phenomenon of Zolpidem addiction is prevalent and can occur outside of hospital settings. Therefore, enhancing the regulation of Zolpidem prescription and enforcing penalties for illegal distribution is warranted. Additionally, health education concerning Zolpidem addiction, early intervention for high-risk populations, and proactive management of complications are also crucial. Intervention strategies should encompass bio-psycho-social factors for optimal effectiveness.

## 4. Strengths and limitations

Inspired by our case, we reviewed similar cases reported in recent years and noted that numerous reports have described the euphoric effects of Zolpidem, suggesting it is a primary contributor to addiction. Based on the available literature, we speculate that the euphoric effect of Zolpidem may be linked to the GABA_A_ receptor. Additionally, we reviewed various approaches to detoxification from Zolpidem. Furthermore, there have been few reports of Zolpidem addiction within the Tibetan population.

Although we observed that the euphoric effect might be associated with Zolpidem addiction at high doses, the precise pathophysiological mechanisms underlying this addiction remain unclear. Further research in both animal and human studies is needed to evaluate the indications and safety of Zolpidem, particularly at doses exceeding 10 mg per day.

## 5. Conclusion

We speculate that Zolpidem addiction is likely linked to its euphoric effects as well as patient characteristics, such as sensation-seeking behavior. A comprehensive approach, involving bio-psycho-social interventions, is essential for an effective detoxification strategy for Zolpidem addiction.

## Acknowledgments

The authors would like to thank the patient for her cooperation.

## Author contributions

**Conceptualization:** Yuanyuan Li.

**Funding acquisition:** Yuanyuan Li.

**Project administration:** Yuanyuan Li.

**Writing – original draft:** Fangfei Xie, Bo Liu.

**Writing – review & editing:** Liqiu Yang, Junqiang Huang, Bin Li, Yuanyuan Li.
